# Between hype and hard evidence: Are large language models ready for implementation in surveillance colonoscopy?

**DOI:** 10.1055/a-2604-7345

**Published:** 2025-06-17

**Authors:** Marco Bustamante-Balén

**Affiliations:** 1 16273 Gastrointestinal Endoscopy Unit, Gastrointestinal Endoscopy Research Group, IIS Hospital La Fe, Hospital Universitari i Politecnic La Fe, Valencia, Spain

**Keywords:** Endoscopy Lower GI Tract, Polyps/adenomas/..., Colorectal cancer, Quality and logistical aspects, Quality management



Colorectal cancer (CRC) is the third most common cancer diagnosed in both sexes in the United States, with an estimate of more than 150,000 cases in 2025 1 . Since the adoption of population-based screening programs, overall incidence of CRC has decreased steadily 2 . More than 15 million colonoscopies are performed annually in the United States, and about 20% of those are surveillance procedures 3 . Several studies have shown that physician adherence to post-polypectomy surveillance guidelines is far from perfect 4 5 , leading to both overutilization and underutilization of colonoscopy. Setting the post-polypectomy surveillance intervals in average-risk adults following guidelines is a repetitive task that could be suitable for artificial intelligence (AI) assistance, thus avoiding biases and deviations from recommendations.

In this issue of Endoscopy International Open, Amini M et al. 6 designed a cross-sectional study aimed at evaluating how effectively two publicly available large language models (LLMs) – ChatGPT 3.5 (GPT3.5) and Bard – could recommend follow-up intervals after colonoscopy, compared with an expert panel. Endoscopy and pathology reports from 549 patients from two different hospitals (a safety-net institution and a tertiary private medical center) were fed into the two LLMs, which then generated recommended follow-up intervals based on the 2020 US Multi-Society Task Force (USMSTF) guidelines. The authors recorded the accuracy of these recommendations relative to endoscopists’ guideline-based consensus. The main finding was that GPT3.5 produced guideline-concordant recommendations in 60.4% of cases overall, significantly outperforming Bard, which correctly matched in only 50.0% of cases. Notably, GPT3.5 maintained comparable performance between the safety net and the private hospital populations, whereas Bard’s accuracy dropped markedly in the safety-net setting (54.3% to 45.7%). Overall concordance of the LLMs with the guideline panel was fair at best (Fleiss’ kappa: GPT 3.5 = 0.324; Bard = 0.219). Both LLMs tended to suggest earlier or later surveillance than recommended in certain complex cases, such as those involving multiple or advanced adenomas. This could have relevant clinical consequences in terms of shortened intervals leading to overutilization of colonoscopy or prolonged intervals increasing risk of missed advanced neoplasia.

Given the findings of this study, what could be the potential role of AI in assisting with the setting of post-polypectomy surveillance intervals? Initially, the results appear discouraging, because the accuracy of the AI models in recommending appropriate intervals was only slightly better than random chance. However, to interpret these results correctly, it is necessary to have some basic knowledge of how LLMs work.

A LLM is an advanced AI system trained on extensive datasets to understand and generate human-like text. Utilizing deep learning techniques, particularly transformer architectures (a specific type of neural network design), LLMs can perform tasks such as translation, summarization, and content creation by predicting and generating text based on input data 7 . A LLM needs training that is, broadly speaking, performed in two phases: 1) pre-training: unsupervised learning on a vast amount of text data; and 2) supervised fine-tuning: several rounds of human interaction with examples to refine the modelʼs ability to generate appropriate responses, with reinforcement of the best responses. In this fine-tuning phase, the model can be trained in specific tasks using more specialized data sets.

Several factors can influence the quality of responses. First, how the LLM has been trained. GPT 3.5 and Bard are previous versions of ChatGPT and Gemini, from Open-AI and Google, respectively, trained with a significantly lower amount of data and with less complex fine-tuning than their more advanced counterparts. This could explain the low concordance in surveillance intervals with the expert panel, because ChatGPT 4.0 has been shown to perform better than GPT3.5 or Bard in several medical contexts 8 9 . Moreover, despite providing the model with a specific source (e.g. the USMSTF 2020 guideline), its performance can be influenced by all its previous training datasets, reducing the consistency of responses to the same questions 10 . This problem may be exacerbated when the variables or nuances of the clinical problem increase, explaining the difficulties of both LLMs when handling familiar history or the number of polyps. It is possible that AI models specifically developed or fine-tuned for medical applications (like Med-PaLM, or Me-LLaMA) could demonstrate improved performance in clinical settings, capturing the nuances of medical decision-making.

Another major influence on LLM efficacy is the input they receive. How the prompt is designed may significantly change the output, affecting consistency of the response. This has led to development of prompt engineering, aimed at providing tools to craft clear, structured, and specific instructions for AI 11 . There are several techniques for prompt engineering, but the authors seem to have used an iterative refinement technique, systematically improving prompts through repeated testing and adjustment. We do not know if the application of other techniques such as few-shot prompting, in which the researchers provide example responses to guide the model, could have changed the results, but some evidence exists indicating that using an advanced version of LLMs has a greater influence than prompt engineering 12 . The other side of the input is the clinical information the researchers added to the prompt. How this information is structured could influence consistency of the output 13 , and could be responsible, in part, for the different performance found between the safety net and the hospital private centers, one using structured reports and the other using free-text documentation.

In the last year, several studies have suggested that AI may not be the disruptive technology that the initial results promised. For instance, studies in real practice using computer-aided diagnosis for polyp characterization could not replicate results of experimental tests. Are we entering the “trough of disillusionment” of the Gartner Hype Cycle? To the contrary, the most plausible explanation is that the storming development of AI (as an example, both LLMs used in the Amini et al. study are currently outdated) prompts us to practical applications without enough preparation. We are facing important new challenges derived from human-–AI relationships, with many nuances influencing the AI responses, nuances we are just learning about. To understand how to integrate AI into our daily practice, which areas are best for seeking assistance from AI, and what can we expect from this technology, we must familiarize ourselves with the processes underlying the final output. Although LLMs may not be ready for immediate application in daily practice, we can only learn by doing, and studies such as the one by Amini et al. highlight the importance of further research into how AI can be effectively used in clinical settings.
